# Carotenoid Pigment Content in Durum Wheat (*Triticum turgidum* L. var *durum*): An Overview of Quantitative Trait Loci and Candidate Genes

**DOI:** 10.3389/fpls.2019.01347

**Published:** 2019-11-07

**Authors:** Pasqualina Colasuonno, Ilaria Marcotuli, Antonio Blanco, Marco Maccaferri, Giuseppe Emanuele Condorelli, Roberto Tuberosa, Roberto Parada, Adriano Costa de Camargo, Andrés R. Schwember, Agata Gadaleta

**Affiliations:** ^1^Department of Agricultural and Environmental Science (DISAAT), University of Bari “Aldo Moro”, Bari, Italy; ^2^Department of Agricultural and Food Sciences (DISTAL), University of Bologna, Bologna, Italy; ^3^Facultad de Agronomía e Ingeniería Forestal, Pontificia Universidad Católica de Chile, Santiago, Chile

**Keywords:** durum wheat, grain yellow pigment content, carotenoids, yellow index, marker-assisted selection

## Abstract

Carotenoid pigment content is an important quality trait as it confers a natural bright yellow color to pasta preferred by consumers (whiteness vs. yellowness) and nutrients, such as provitamin A and antioxidants, essential for human diet. The main goal of the present review is to summarize the knowledge about the genetic regulation of the accumulation of pigment content in durum wheat grain and describe the genetic improvements obtained by using breeding approaches in the last two decades. Although carotenoid pigment content is a quantitative character regulated by various genes with additive effects, its high heritability has facilitated the durum breeding progress for this quality trait. Mapping research for yellow index and yellow pigment content has identified quantitative trait loci (QTL) on all wheat chromosomes. The major QTL, accounting for up to 60%, were mapped on 7L homoeologous chromosome arms, and they are explained by allelic variations of the phytoene synthase (*PSY*) genes. Minor QTL were detected on all chromosomes and associated to significant molecular markers, indicating the complexity of the trait. Despite there being currently a better knowledge of the mechanisms controlling carotenoid content and composition, there are gaps that require further investigation and bridging to better understand the genetic architecture of this important trait. The development and the utilization of molecular markers in marker-assisted selection (MAS) programs for improving grain quality have been reviewed and discussed.

## Introduction

Durum wheat (*Triticum turgidum* L. ssp. *durum*) is a cereal crop grown around the world on about 17 million hectares and with about 37 million tons produced annually during the last decade, with wide variation from 32 to 42 million tons caused mainly by drought and heat stresses (data FAO, 2017). Globally durum wheat represents only 8% of the whole area cultivated with wheat and about 5% of world wheat production. The principal durum-producing countries are the European Union, Canada, Turkey, USA, Algeria, Kazakhstan, and Mexico, whereas minor production countries encompass Syria, Morocco, Tunisia, India, Australia, and Argentina and Chile, among others. Major producers in the EU are Italy, France, Greece, and Spain. Although durum wheat is a relatively minor crop worldwide, it is the main crop for many areas of the Mediterranean basin and makes up the raw material for various finished products such as pasta and couscous consumed all over the world (Kabbaj et al., 2017).

Grain protein content and conformation together with yellow color are the most valued wheat quality traits, which are important in the commercial, nutritional, and technological values of grain and end products of both common and durum wheat (Sissons, 2008; Nazco et al., 2012; Subira et al., 2014; Mazzeo et al., 2017).

Semolina and pasta color are the consequence of two distinct constituents: yellow (desirable) and brown (undesirable) pigments. The yellow color, principally explained by carotenoid accumulation in kernels, has been considered a source of significant nutrients/antioxidant compounds and a factor for the commercial value since consumers prefer a bright yellow color of semolina and the pasta products.

It is a typical quantitative trait controlled by a complex genetic system (quantitative trait loci, QTL) and influenced by environmental factors. As confirmed by the high value of heritability, the genetic component is predominant, and this has facilitated the success of breeding programs (Elouafi et al., 2001). A consequence of this intense breeding activity has been proved by the higher carotenoid concentration in durum wheat cultivated varieties compared to the wild ones (Digesù et al., 2009).

Genetic analyses based on molecular markers have mapped major QTL for carotenoid content on homoeologous group 7. Minor QTL, associated to significant markers, were detected on almost all chromosomes of the durum wheat genome. Significant marker-trait associations for carotenoid content have been detected on all of the chromosomes by linkage mapping. Association mapping has been used as a new strategy for the dissection of this trait and highlighted its complexity.

Due to the importance of this quality trait, the aim of the present review is to summarize the information available on the detection of QTL for carotenoid content and individual components and the identification of candidate genes in durum wheat.

## Carotenoids and Nutritional Aspects

In the first years of the 21st century, major breeding programs were focused on improving the durum productivity traits of wheat, such as grain yield and biotic and abiotic stress resistance. Recently, the attention on food quality over quantity has switched the research aims at increasing wheat nutritional value estimated through different parameters, like protein content, water absorption, and flour color. The latter one is due to the carotenoid pigments, whose nutritional benefits in human health is worldwide recognized (Sommer and Davidson, 2002).

Over 600 carotenoids have been identified in plants and microorganisms. They are one of the most studied groups of natural pigments, because of their broad distribution, structural variety, and multiple functions. All fruit and plant color, ranging from yellow to red, are good sources of carotenoids (Britton, 1998).

Bendich and Olson were the first scientists that characterize more than 750 carotenoid compounds (Bendich and Olson, 1989; Olson and Krinsky, 1995; DellaPenna and Pogson, 2006). *Carotenoids* is the generic term indicating the majority of red, orange, and yellow pigments naturally encountered in photosynthetic organisms and in certain fungi and bacteria (Britton 1995; Khoo et al., 2011).

Most carotenoids are tetraterpenoids (C40 compounds), which are composed of eight isoprenic units linked in a linear and symmetrical structure. The basic cyclic structure can be changed by dehydrogenation, hydrogenation, cyclization, and oxidation reactions, while the high chemical reactivity has been induced by a complex system of double bonds (Oliver and Palou, 2000). Two classes of carotenoids are found in nature: (a) the carotenes, linear tetraterpenoid hydrocarbons (i.e., β-carotene) that can be cyclized at one or both ends of the molecule, and (b) the xanthophylls, composed by one or more oxygen groups (i.e., lutein, violaxanthin, neoxanthin, and zeaxanthin) (Van den Berg et al., 2000). Overall, carotenoids possess general properties common to all carotenoids (i.e., antioxidant nature) from specific ones (i.e., provitamin A, only particular ones) (Young and Lowe, 2018).

Food, animal feed, and pharmaceutical industries are using carotenoids for their color properties (in fruit juices, pasta, candies, cheese, chicken skin, and egg yolk) or for their contribution in the flavor and fragrances of some foods (Landrum, 2009). In addition, in a context of increasing interest in improving health through the consumption of natural products, they are considered also in food fortification. It has been demonstrated that they are precursors of vitamin A, generating health advantages, such as antioxidant properties, reinforcing the immune system, decreasing the risk of degenerative and cardiovascular ailments, anti-obesity/hypolipidemic properties, and defense of the macula region of the retina (Mezzomo et al., 2015).

Among the carotenoid compounds, following the presence/absence of the provitamin A in the molecule structure, fewer than 10% show a significant biological activity and act as vitamin A precursors. The precursors of vitamin A have a minimum of one β-ionone ring and a polienic chain with 11 carbons at least (Kelly et al., 2018).

Bioactive compounds and vitamin A are categorized as antioxidants, playing a crucial function in humans’ health such as in growth, in the development and maintenance of epithelial tissues, in the immune system strength, and in the first protection mechanism against oxidative stress. Antioxidants reduce the singlet oxygen in the human body and scavenge free radicals, i.e., reactive nitrogen species (RNS) and reactive oxygen species (ROS) (Choe and Min, 2005; Leong et al., 2018). The oxidation of carotenoids by ROS causes the loss of their characteristic color inducing cell protection and the prevention of degenerative diseases (Halliwell, 2011; Guaadaoui et al., 2014).

Carotenoids are transformed to vitamin A to satisfy the body requirements, changing the levels of conversion efficiency (Mezzomo and Ferreira, 2016). A prolonged deficiency of vitamin A can produce skin modifications, corneal ulcers, and night blindness, while a surplus is toxic and may be associated to congenital malformation in pregnancy, bone diseases in patients with chronic renal malfunction, blindness, xerophthalmia, and death (Mezzomo et al., 2015).

According to the high unsaturation degree of carotenoids, light, heat, acids, and enzymatic oxidation can change their structure from the trans-isomers (the most stable type in nature) to the cis-structure, producing a minor decrease of color and provitamin activity (Schroeder and Johnson, 1995).

## The Carotenoids’ Value in Yellow Color of Wheat Products

The yellow-amber color of semolina is caused by the carotenoid (yellow) pigment content (YPC) in the entire grain, which is known as the yellow index (YI) of semolina at a commercial level (CIE, 1986). The average carotenoid concentration in durum wheat is 6.2 ± 0.13 mg/kg in dry weight, determining the pasta color (Beleggia et al., 2011; Brandolini et al., 2015).

In wheat kernel, a wide range of carotenoids have been detected such as lutein, β-carotene, zeaxanthin, β-cryptoxanthin, β-apocarotenal, antheraxanthin, taraxanthin (lutein-5,6-epoxide), flavoxanthin, and triticoxanthin (Lachman et al., 2017).

The pigments are variable: α- and β-carotene (7.7%) are mainly located in the germ, while lutein, the most abundant pigment (86–94%) (Konopka et al., 2004; Digesù et al., 2009), is equally distributed across the layers (Borrelli et al., 2008). Specifically, aleurone layer, starchy endosperm, and germ contain 0.425, 0.557, and 2.157 mg/kg of lutein, respectively. In parallel, aleurone and germ contain 0.776 and 3.094 mg/kg zeaxanthin.

During the milling process, a large amount of these components are gradually reduced, depending on the extraction rate (Paznocht et al., 2019). Lutein, and a small amount of zeaxanthin, has higher cooking stability compared to other carotenoids commonly present in foods, for example, β-cryptoxanthin and β-carotene (Britton and Khachik, 2009; Kean et al., 2011).

In the wheat end products, steady-state level of carotenoids is dependent on the equilibrium between biosynthesis and degradation in the processing phase. This last process has been principally attributed to oxidative enzymes, such as the lipoxygenases (LOX), polyphenol oxidase (PPO), and peroxidases (PER), that can cause the loss of yellow color of flour and pasta (Hessler et al., 2002; Leenhardt et al., 2006; Garbus et al., 2013; Morris, 2018).

## Carotenoid Biosynthesis and Degradation Pathways

**Figure 1:**
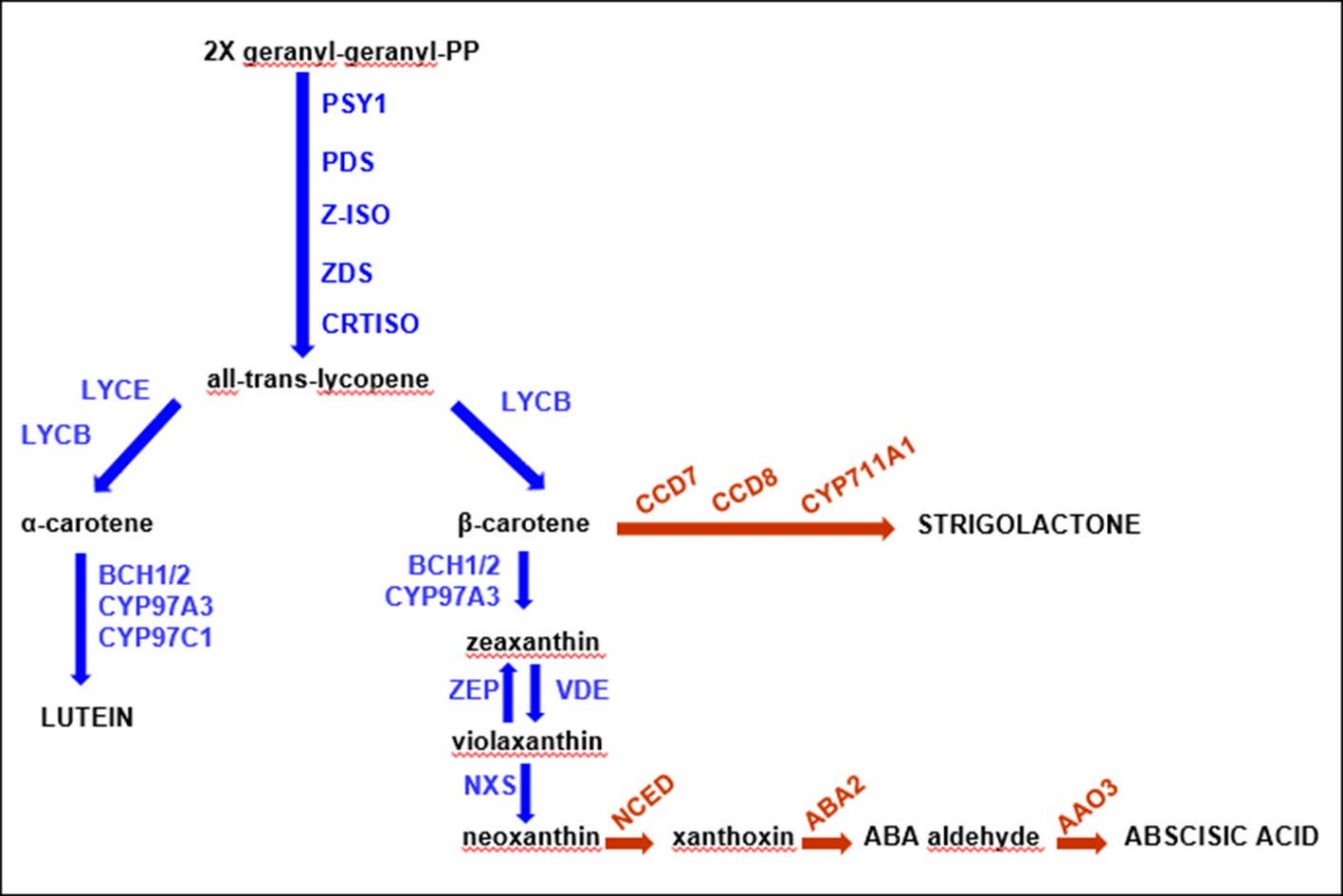
Schematic carotenoid pathway. The main components of the biosynthetic chain have been reported in the figure in black font, while all the enzymes involved are blue. The proteins from the related pathways are indicated in red.

The carotenoid metabolic biosynthetic pathway has been thoroughly investigated in some plants, including *Arabidopsis*, rice, maize, pepper, tomato, and orange (see studies by Colasuonno et al., 2017a; Rodriguez-Concepcion et al., 2018; Sun et al., 2018). This biosynthetic route has been examined in depth (**Figure 1**), and all genes and enzymes involved have been isolated and well characterized. It starts with the condensation of two molecules of geranylgeranyl diphosphate (GGPP) by phytoene synthase (*PSY*) to generate phytoene, which normally is not accumulated in tissues. This step is a key rate-limiting stage of carotenoid biosynthesis, since it might affect the carotenoid pool content (Cazzonelli and Pogson, 2010; Ke et al., 2019). Following a sequence of desaturation and isomerization reactions catalyzed by phytoene desaturase (*PDS*), zeta-carotene isomerase (*Z-ISO*), zeta-carotene desaturase (*ZDS*), and carotenoid isomerase (*CRTISO*), the lycopene biosynthesis, a red-colored carotenoid, takes place. Double lycopene cyclization produces orange β-carotene (branch β-β) or α-carotene (branch β-ε). Further hydroxylation of α-carotene generates yellow zeinoxanthin and lutein, while the modification of β-carotene produces β-cryptoxanthin, zeaxanthin, antheraxanthin, violaxanthin, and neoxanthin (**Figure 1**). These steps are catalyzed by two non-heme β-carotene hydroxylases (*BCH1* and *BCH2*) and two heme hydroxylases (*CYP97A* and *CYP97C*), respectively (Sun et al., 2018). The consequent epoxidation and de-epoxidation of zeaxanthin by zeaxanthin epoxidase (*ZEP*) and violaxanthin de-epoxidase (*VDE*) induce the production of xanthophylls, molecules involved in plant protection’s mechanisms (Jahns and Holzwarth, 2012). The last phase of carotenoid biosynthesis consists in the transformation of violaxanthin into neoxanthin by neoxanthinsynthase (*NXS*) (Sun et al., 2018). Further oxidative cleavage reactions of violaxanthin and neoxanthin produce xanthoxin, transformed to the abscisic acid (ABA) plant hormone by ABA-aldehyde enzymes (Seo and Koshiba, 2002).

Strigolactones are carotenoid derivatives and originated from the functioning of carotenoid cleavage dioxygenases (CCDs), contributing to the regular accumulation and balanced levels of pigments (Al-Babili and Bouwmeester, 2015; Nisar et al., 2015).

Numerous studies focused on all the carotenoid enzymes have shown that the inheritance of this trait in wheat is of quantitative nature, and it is highly heritable (Digesù et al., 2009; Blanco et al., 2011; Roncallo et al., 2012; Schulthess and Schwember, 2013; Schulthess et al., 2013). Consequently, the correct approach to study carotenoid pigments is to conduct a QTL mapping strategy.

## Determination of Yellow Pigments Content and Their Single Components

One of the principal problems of carotenoid analyses is testing for their composition and concentration through reproducible and accurate methods, often using only small amounts of seeds. The detection of carotenoid levels is technically restricted by several limitations such as the interference with some regulation/degradation processes and the product storage (Sandmann, 2001). Carotenoid content is a complex trait, and there are several procedures for measuring total pigment concentration and individual pigment compounds.

The reference methods for the total YPC are Standard Method 152 (ICC, 1990) of the International Association for Cereal Science and Technology (ICC) and AACC International Official Method 14-50.01 (AACC International, 2013). These two procedures rely on the extraction of total pigmwwents in water-saturated n-butanol followed by a spectrophotometric quantification of the absorbance of the alcoholic extract at 435.8 nm (the wavelength of maximum absorption of lutein, the dominant carotenoid in durum wheat), using USA/Canada standards (Fu et al., 2017).

Alternatively, pigment content can be measured by the YI determination based on the quantification of flour light reflectance. Analysis with Minolta CR-300 Chroma Meter (Konica Minolta Pty Ltd, Macquarie Park, NSW) geared up with a pulsed xenon arc lamp is the most used instrument for YI analysis. It provides the brown and yellow indexes, after calibration with standard flour control samples. Specially, the measurements consist of the L* (lightness, ranging from 0 for darkness to 100 for total light), a* (red–green chromaticity), and b* (yellow–blue chromaticity) coordinates of the Munsell color system, employing D65 lightning (CIE, 1986). The b* value is directly linked to the lutein and carotene contents, determining the variation in the yellow intensity (Rodriguez-Amaya and Kimura, 2004). Near-infrared (NIR) spectroscopy is a fast, non-destructive, and economic technique, useful for prediction of individual carotenoid pigments in maize and wheat semolina (McCaig et al., 1992).

Accurate measurements of carotenoids and their components can be exclusively obtained by high-performance liquid chromatography (HPLC) analysis (Brandolini et al., 2008; Fu et al., 2017). HPLC, with grade solvents of methyl tert-butyl ether and methanol, and photodiode array detector, allows the identification and separation of carotenoid compounds (Panfili et al., 2004). Detailed HPLC protocols to identify carotenoids in cereals and end products have been designed by several research groups (Burkhardt and Böhm, 2007; Digesù et al., 2009; Irakli et al., 2011; Shen et al., 2015).

## Qtl Detection in Durum Wheat

YPC is controlled by various genes with additive effects that are affected by different environmental conditions (Schulthess et al., 2013). In classic quantitative genetics research, the creation of linkage maps in biparental populations allowed studying the number of loci regulating the trait, the phenotypic and pleiotropic effects, as well as the epistatic interactions with other QTL, enabling the identification and characterization of candidate genes. Mapping studies for YPC and YI in various biparental populations have led to QTL detection across all wheat chromosomes (**Table 1**).

**Table 1:** Summary of quantitative trait loci (QTL) clusters for grain yellow pigment content (YPC) and yellow index (YI) reported in tetraploid wheat from the literature and from this study.

Chr.	Marker, marker interval *	Map position (cM)**	Carotenoid trait	QTL type***	R2 (%)****	Plant material	References
1AS	hap_1A_1	1.3–1.7	YPC	GWAS	8.5	Canadian durum breeding lines (192 lines)	N’Diaye et al., 2018
1AS	gwm136-1A	0–4.6	YPC	BP	12.3	Colosseo × Lloyd (176 RILs)	This study
1AS	hap_1A_3	58.4–61.2	YPC	GWAS	7	Canadian durum breeding lines (192 lines)	N’Diaye et al., 2018
1AS	barc83-gwm135^¤^	47.8–52.8–57.8	YPC	GWAS	NA	Worldwide elite durum wheat collection (93 lines)	Reimer et al., 2008
1AS	IWB72019	51–59.7–68.4	YPC	BP	5.5	Meridiano × Claudio (181 RILs)	This study
1AL	hap_1A_6	149.5–149.5	YI	GWAS	3.8	Canadian durum breeding lines (192 lines)	N’Diaye et al., 2018
1AL	barc158-barc17^¤^	146.4–151.4–156.4	YPC	GWAS	NA	Worldwide elite durum wheat collection (93 lines)	Reimer et al., 2008
1BS	wPt-2694	22.2–27.2–32.5	YI	GWAS	6	Landraces (72 lines), modern cultivars (20 lines)	Rosello et al., 2018
1BS	barc137-wmc626^¤^	29.6–36.7–41.7	YI	GWAS	3.8	Canadian durum breeding lines (192 lines)	N’Diaye et al., 2018
1BS	hap_1B_3	35.7–38.8	YPC	GWAS	12.7	Canadian durum breeding lines (192 lines)	N’Diaye et al., 2018
1BL	BE443797_436–barc302	38–60	YPC, YI	BP	9	UC1113 × Kofa (93 RILs)	Zhang et al., 2008
1BL	BE443797_436–barc302	38–60	YI	BP	10.8	UC1113 × Kofa (93 RILs)	Roncallo et al., 2012
1BL	IWB73028-IWB27784	103.5–(106–110)–113.5	YPC	BP	19.3	Svevo × Ciccio (120 RILs)	Colasuonno et al., 2014
1BL	hap_1B_6	109–109.8	YPC	GWAS	5.2	Canadian durum breeding lines (192 lines)	N’Diaye et al., 2018
1BL	hap_1B_7	115.7–119.1	YPC	GWAS	13.8	Canadian durum breeding lines (192 lines)	N’Diaye et al., 2018
1BL	Cap3_137325-1B, IWB6947, IWB73429	115.2–117.4–118.6	YPC	BP	2.8	Colosseo × Lloyd	This study
1BL	gwm268-1B	106.8–118.9–131.0	YPC	BP	2.2	Kofa × Svevo (249 RILs)	This study
1BL	wmc44	135.6–140.6–145.6	YPC	GWAS	NA	Worldwide elite durum wheat collection (93 lines)	Reimer et al., 2008
1BL	IWB435^¤^	145.3–156.3–161.3	YPC	BP	4	Colosseo × Lloyd	This study
2AS	gwm1115-2A	53.2–69.9–86.6	YPC	BP	2.3	Kofa × Svevo	This study
2AS	IWB77592-IWB79691	74.6–78	YPC, YI	GWAS	15.1	Canadian durum wheat collection (169 lines)	N’Diaye et al., 2017
2AS	gwm425	99	YPC, YI	BP	NA	W9262-260D3 × Kofa (155 RILs)	Pozniak et al., 2007
2AS×	wmc522-wmc296-gwm425-gwm95	63.6–105.6	YPC	GWAS	NA	Worldwide elite durum wheat collection (93 lines)	Reimer et al., 2008
2AS	gwm425-gwm372^¤^	99–107.7	YPC, YI	BP	11.8–24.5	Latino Primadur (121 RILs)	Blanco et al., 2011
2AS	IWB72639	102.7–107.7–112.7	YPC	BP	3.4–13.5	Svevo × Ciccio (120 RILs)	Colasuonno et al., 2014
2BL	IWB73809^¤^	90–(94–113)–117	YPC	BP	16.3–16.4	Svevo × Ciccio (120 RILs)	Colasuonno et al., 2014
2BL	IWB73809	108.3–112.3–118.3	YPC	BP	16.3–16.4	Svevo × Ciccio (120 RILs)	Colasuonno et al., 2014
3BS	gwm389	0-5–6	YPC	GWAS	NA	Worldwide elite durum wheat collection (93 lines)	Reimer et al., 2008
3BS	CS-ssr7-3B	5.4–12.1–18.8	YPC	BP	4.8	Kofa × Svevo	This study
3BS	wPt-8140	42.8–47.8–52.8	YI	GWAS	7	Landraces (72 lines), modern cultivars (20 lines)	Rosello et al., 2018
3BS	wmc505-3B^¤^	48.6–63.3–78.0	YPC	BP	1.9	Kofa × Svevo	This study
3BL	wPt-4401-3B, IWB71566	170.9–183.8–196.6	YPC	BP	2	Colosseo × Lloyd	This study
3BL	barc77-gwm299	170.7–175.7–197.1	YPC	GWAS	NA	Worldwide elite durum wheat collection (93 lines)	Reimer et al., 2008
3BL	gwm299-barc84	192–198	YPC, YI	BP	11.5–16.2 (YPC), 9.4–16.6 (YI)	Latino × Primadur (121 RILs)	Blanco et al., 2011
3BL	IWB45539-IWB58810	200.5–205.5	YPC, YI	GWAS	13.4	Canadian durum wheat collection (169 lines)	N’Diaye et al., 2017
3BL	wmc632-gwm340	206.9–211.9	YPC	GWAS	NA	Worldwide elite durum wheat collection (93 lines)	Reimer et al., 2008
3BL	IWB8780-IWB72417	208–209.6	YPC, YI	GWAS	14.6	Canadian durum wheat collection (169 lines)	N’Diaye et al., 2017
3BL	wPt-2416	211.3–216.3–221.3	YI	GWAS	5	Landraces (72 lines), modern cultivars (20 lines)	Rosello et al., 2018
4AL	Lpx-A3-gwm192-wmc617^¤^	63.6–81	YPC, YI	BP	10.6–12	UC1113 × Kofa (93 RILs)	Roncallo et al., 2012
4AL	hap_4A_2	64.1–64.1	YPC	GWAS	5.5	Canadian durum breeding lines (192 lines)	N’Diaye et al., 2018
4AL	wPt-0162	69.7	YI	GWAS	5	Landraces (72 lines), modern cultivars (20 lines)	Rosello et al., 2018
4AL	dupw4-barc170	88.9–90.4	YI	BP	8.4–9.1	UC1113 × Kofa (93 RILs)	Roncallo et al., 2012
4AL	barc170	85.4–90.4–95.4	YPC	GWAS	NA	Worldwide elite durum wheat collection (93 lines)	Reimer et al., 2008
4AL	hap_4A_3	90.6–90.6	YPC	GWAS	3.6	Canadian durum breeding lines (192 lines)	N’Diaye et al., 2018
4AL	barc327-gwm160-barc52^¤^	160–172	YPC	BP	5	UC1113 × Kofa (93 RILs)	Zhang et al., 2008
4AL	wmc219-psr573.2	165.2–167.8	YPC	BP	6.7–12.0	UC1113 × Kofa (93 RILs)	Roncallo et al., 2012
4AL	wmc219-4A	158.3–165.2–172.1	YPC	BP	4.1	Kofa × Svevo	This study
4AL	hap_4A_6	173.3–175.8	YPC	GWAS	8	Canadian durum breeding lines (192 lines)	N’Diaye et al., 2018
4AL	hap_4A_7	173.3–175.9	YI	GWAS	6.8	Canadian durum breeding lines (192 lines)	N’Diaye et al., 2018
4BS	gwm368-barc20	34.4–39.4–41.3–46.3	YPC	GWAS	NA	Worldwide elite durum wheat collection (93 lines)	Reimer et al., 2008
4BS	IWB72011^¤^	38.9–43.9–48.9	YPC, YI	GWAS	11.1	Durum collection (124 lines)	Colasuonno et al., 2017a
4BS	hap_4B_2	59.8–60.4	YI	GWAS	13.3	Canadian durum breeding lines (192 lines)	N’Diaye et al., 2018
4BS	hap_4B_2	59.8–60.4	YPC	GWAS	6.8	Canadian durum breeding lines (192 lines)	N’Diaye et al., 2018
4BS	IWB70599	58.7–60.4	YPC, YI	GWAS	14.1	Canadian durum wheat collection (169 lines)	N’Diaye et al., 2017
4BS	gwm495	60–65	YPC, YI	BP	NA	W9262-260D3 × Kofa (155 RILs)	Pozniak et al., 2007
4BL	IWB73624	88.7–95.1–101.5	YPC	BP	7.1	Colosseo × Lloyd	This study
4BL	hap_4B_4	105.5–110.2	YPC	GWAS	9.1	Canadian durum breeding lines (192 lines)	N’Diaye et al., 2018
5AS	hap_5A_1	43.8–43.8	YPC	GWAS	4.8	Canadian durum breeding lines (192 lines)	N’Diaye et al., 2018
5AS	gwm293-gwm304	41.9–46.9–51.9	YPC	GWAS	NA	Worldwide elite durum wheat collection (93 lines)	Reimer et al., 2008
5AS	gwm304-IWB73092^¤^	45–60	YPC, YI	BP	9.4–17.9 (YPC), 7.4–16.4 (YI)	Latino × Primadur (121 RILs)	Blanco et al., 2011
5AS	hap_5A_2	50.5–54.9	YPC	GWAS	5.7	Canadian durum breeding lines (192 lines)	N’Diaye et al., 2018
5AS	IWB73092	54.2–59.2–64.2	YPC	BP	12.6	Svevo × Ciccio (120 RILs)	Colasuonno et al., 2014
5AS	IWB12396	53.9–68.2–82.5	YPC	BP	3.1	Meridiano × Claudio	This study
5AS	hap_5A_3	64.2–73.6	YPC	GWAS	15.3	Canadian durum breeding lines (192 lines)	N’Diaye et al., 2018
5BL	wmc790-cfa2019	90.4–100.4	YPC	GWAS	NA	Worldwide elite durum wheat collection (93 lines)	Reimer et al., 2008
5BL	gwm499-BE495277_399	90–94	YPC, YI	BP	9–12.2	UC1113 × Kofa (93 RILs)	Roncallo et al., 2012
5BL	gwm408-barc232^¤^	140.3–146.3	YPC	BP	7.3	PDW 233 × Bhalegaon 4 (140 RILs)	Patil et al., 2008
5BL	tPt-1253	142–145–148	YPC	BP	21.8	Svevo × Ciccio (120 RILs)	Colasuonno et al., 2014
5BL	cfd86-wmc508	152.5–157.5–162.5	YPC	GWAS	NA	Worldwide elite durum wheat collection (93 lines)	Reimer et al., 2008
5BL	311891-5B, IWB70782, IWB63422	139.7–152.5–165.3	YPC	BP	3	Colosseo × Lloyd	This study
5BL	wPt-8125-5B	145.5–154.5–163.5	YPC	BP	6.2	Meridiano × Claudio	This study
6AS	gwm334	0–4.3–9.3	YPC	GWAS	NA	Worldwide elite durum wheat collection (93 lines)	Reimer et al., 2008
6AS	wPt-8443	0–10	YI	GWAS	5	Landraces (72 lines), modern cultivars (20 lines)	Rosello et al., 2018
6AS	hap_6A_3	39.5–41	YPC	GWAS	9.7	Canadian durum breeding lines (192 lines)	N’Diaye et al., 2018
6AS	hap_6A_4	39.5–41	YI	GWAS	5.4	Canadian durum breeding lines (192 lines)	N’Diaye et al., 2018
6AL	Cap3_141157-6A, GWM132, IWB71882	50.8–52.5–54.2	YPC	BP	18.8	Colosseo × Lloyd	This study
6AL	barc146	47.552.5–57.5	YPC	GWAS	NA	Worldwide elite durum wheat collection (93 lines)	Reimer et al., 2008
6AL	barc146-gwm132	53–63	YPC, YI	BP	16.1–21.4	UC1113 × Kofa (93 RILs)	Roncallo et al., 2012
6AL	barc1077-6A	54.7–57.7–60.7	YPC	BP	9.4	Kofa × Svevo	This study
6AL	barc113-gwm570-wmc553	73–95	YPC, YI	BP	14	UC1113 × Kofa (93 RILs)	Zhang et al., 2008
6AL	barc113-wmc553^¤^	73.6–95	YI	BP	29 (YPC), 17.9 (YI)	UC1113 × Kofa (93 RILs)	Roncallo et al., 2012
6AL	IWB14365	88.4–93.4–98.4	YI	GWAS	NA	Durum collection (124 lines)	Colasuonno et al., 2017a
6AL	barc353-gwm169	97.6–114	YPC, YI	BP	9.8–12.4	UC1113 × Kofa (93 RILs)	Roncallo et al., 2012
6AL	gwm169-BE483091_472	114–124	YI	BP	10.4	UC1113 × Kofa (93 RILs)	Roncallo et al., 2012
6BL	gwm193	69.9–74.9–79.9	YPC	GWAS	NA	Worldwide elite durum wheat collection (93 lines)	Reimer et al., 2008
6BL	gwm193-wmc539^¤^	74–90	YPC, YI	BP	NA	W9262-260D3 × Kofa (155 RILs)	Pozniak et al., 2007
6BL	hap_6B_5	92.3–96	YPC	GWAS	6	Canadian durum breeding lines (192 lines)	N’Diaye et al., 2018
7AS	gwm471	0–0.3–5.3	YPC	GWAS	NA	Worldwide elite durum wheat collection (93 lines)	Reimer et al., 2008
7AS	wmc168-barc219^¤^	1–5	YI	BP	12.6	UC1113 × Kofa (93 RILs)	Roncallo et al., 2012
7AS	hap_7A_1	21.9–21.9	YPC	GWAS	4.6	Canadian durum breeding lines (192 lines)	N’Diaye et al., 2018
7AS	hap_7A_3	55.9–62.5	YPC	GWAS	21.3	Canadian durum breeding lines (192 lines)	N’Diaye et al., 2018
7AS	barc127-cfa2028	50.4–55.4–60.4	YPC	GWAS	NA	Worldwide elite durum wheat collection (93 lines)	Reimer et al., 2008
7AS	IWB8374	56.6–61.6–66.6	YI	GWAS	12.6	Durum collection (124 lines)	Colasuonno et al., 2017a
7AS	hap_7A_4	82.4–84.4	YPC	GWAS	8.8	Canadian durum breeding lines (192 lines)	N’Diaye et al., 2018
7AS	BQ170462_176-barc174	85–90–95	YPC, YI	BP	11.7	UC1113 × Kofa (93 RILs)	Roncallo et al., 2012
7AS	hap_7A_5	90.9–90.9	YPC	GWAS	3.4	Canadian durum breeding lines (192 lines)	N’Diaye et al., 2018
7AL	gwm1065-7A^¤^	88.1–90.9–93.7	YPC	BP	10	Kofa × Svevo	This study
7AL	IWB72567	97.3–102.3–107.3	YPC, YI	GWAS	18.4	Durum collection (124 lines)	Colasuonno et al., 2017a
7AL	IWB11003	98–102.3–106.6	YPC	BP	11.2	Meridiano × Claudio	This study
7AL	barc108-wmc283-wmc603	107.8–113.4–118.4	YPC	GWAS	NA	Worldwide elite durum wheat collection (93 lines)	Reimer et al., 2008
7AL	hap_7A_7	112.2–118	YPC	GWAS	9.4	Canadian durum breeding lines (192 lines)	N’Diaye et al., 2018
7A	gwm276-cfd6	144.8–145.5	YPC	BP	22	UC1113 × Kofa (93 RILs)	Zhang and Dubcovsky, 2008
7AL	gwm276-wmc116-cfd6	144.8–145.5	YPC	BP	6.3 (YPC), 9.8–22.5 (YI)	UC1113 × Kofa (93 RILs)	Zhang et al., 2008
7AL	wmc116-cfd6	144.8–145.5	YI	BP	9.8–22.5	UC1113 × Kofa (93 RILs)	Roncallo et al., 2012
7AL	gwn282-IWB59875^¤^	170–183	YPC, YI	BP	19.8–30.4 (YPC), 13–15.7 (YI)	Latino × Primadur (121 RILs)	Blanco et al., 2011
7A	IWB59875	177.3–180.3–183.3	YPC	BP	51.6	Svevo × Ciccio (120 RILs)	Colasuonno et al., 2014
7AL	IWB72397	180.2–181.8	YPC, YI	GWAS	35.6	Canadian durum wheat collection (169 lines)	N’Diaye et al., 2017
7AL	IWB59875	175.3–180.3–185.3	YI	GWAS	12.2	Durum collection (124 lines)	Colasuonno et al., 2017a
7AL	IWB28063	179.5–181.8–184.1	YPC	BP	19.5	Meridiano × Claudio	This study
7AL	wgwm63-gwm282^¤^	192–206	YPC	BP	NA	Omrabi5 × *Triticum dicoccoides* 600545 114 RILs	Elouafi et al., 2001
7AL	Xscar3362^¤^	192–206	YPC	BP	22.6–55.2	PDW 233 (YAV’S’/TEN’S’) × Bhalegaon 4 140 RILs	Patil et al., 2008
7AL	*Psy1-A1* ^¤^	192–206	YPC, YI	BP	NA	Commander × DT733 (110 RILs); Strongfield × Blackbird (89 DHs); Strongfield × Commander (106 RILs)	Singh et al., 2009
7AL	*Psy1-A1* ^¤^	192–206	YI	BP	NA	Advanced breeding lines (100 lines, F7–F10 generations)	He et al., 2009
7AL	D_304196-*PsyA1*	192–206	YPC, YI	BP	42–53.2 (YPC), 26.1–32.4 (YI)	Latino × Primadur (121 RILs)	Blanco et al., 2011
7AL	*Psy1-A1*	192–206	YPC,YI	BP	NA	Breeding lines (65), landraces (155 lines)	Campos et al., 2016
7AL	hap_7A_11	193.9–194.6	YPC	GWAS	5.7	Canadian durum breeding lines (192 lines)	N’Diaye et al., 2018
7AL	hap_7A_11	193.9–194.6	YI	GWAS	3.9	Canadian durum breeding lines (192 lines)	N’Diaye et al., 2018
7AL	IWB49295	198.4–203.4–208.4	YPC	GWAS	10.4	Durum collection (124 lines)	Colasuonno et al., 2017a
7BS	wmc546-wmc335	55–73	YPC	BP	8.75	PDW 233 (YAV’S’/TEN’S’) × Bhalegaon 4 140 RILs	Patil et al., 2008
7BS	wmc182-7B	50–51.6–53.2	YPC	BP	19.3	Kofa × Svevo	This study
7BS	Cap3_173782-7B, IWB72147, IWB12844	53.5–58.3–63.1	YPC	BP	11.7	Colosseo × Lloyd	This study
7BS	barc23-barc72-gwm297^¤^	66.3–67.9–72.8	YPC, YI	BP	8.5–12.8	UC1113 × Kofa (93 RILs)	Roncallo et al., 2012
7BL	wmc758-wmc475-gwm333-wmc396	81.2–103.2	YPC	GWAS	NA	Worldwide elite durum wheat collection (93 lines)	Reimer et al., 2008
7BL	Cap3_127025-7B, IWB8805, IWB11767, IWB12371	88.9–104.4–119.8	YPC	BP	4.5	Colosseo × Lloyd	This study
7BL	hap_7B_3	120.4–127.4	YPC	GWAS	11.6	Canadian durum breeding lines (192 lines)	N’Diaye et al., 2018
7BL	hap_7B_3	120.4–127.4	YI	GWAS	10.3	Canadian durum breeding lines (192 lines)	N’Diaye et al., 2018
7BL	wmc311-cfa2257	181.2–185	YPC, YI	BP	7	UC1113 × Kofa (93 RILs)	Zhang et al., 2008
7BL	wmc311-wmc276	181.2–185	YPC	BP	16.9	UC1113 × Kofa (93 RILs)	Roncallo et al., 2012
7BL	wPt-5138	180–189	YI	GWAS	6	Landraces (72 lines), modern cultivars (20 lines)	Rosello et al., 2018
7BL	cfa2040-Psy-B1-barc1073-cfa2257^¤^	181.2–207	YI, YPC	BP	6.6–16.9	UC1113 × Kofa (93 RILs)	Roncallo et al., 2012
7BL	wPt-744987-7B	194.8–202.9–211.0	YPC	BP	4.3	Meridiano × Claudio	This study
7BL	Psy1-B1^¤^	204	YI	BP	NA	100 advanced breeding lines (F7–F10 generations)	He et al., 2009
7BL	Psy1-B1^¤^	204	YPC, YI	BP	NA	W9262-260D3 × Kofa (155 RILs)	Pozniak et al., 2007
7BL	Psy1-B1-wmc10-gwm146	204	YPC	GWAS	NA	Worldwide elite durum wheat collection (93 lines)	Reimer et al., 2008
7BL	ubw18b-7B	196.3–204.9–213.5	YPC	BP	4.5	Kofa × Svevo	This study
7BL	IWB34193-IWB12638	202.9–206.3	YPC, YI	GWAS	9.3	Canadian durum wheat collection (169 lines)	N’Diaye et al., 2017
7BL	gwm344	203	YPC	BP	52	Omrabi5 × *T. dicoccoides* 600545 114 RILs	Elouafi et al., 2001
7BL	barc340-cfa2257	195.9–207	YPC	BP	7	UC1113 × Kofa (93 RILs)	Zhang and Dubcovsky, 2008

*QTL markers are SSR, DART, single nucleotide polymorphism (SNP) markers mapped in the durum wheat consensus map (Maccaferri et al., 2014).

**QTL marker, marker interval: reported by projecting the QTL interval and QTL peak markers onto the durum wheat consensus map (Maccaferri et al., 2014).

***BP = for QTL obtained by using biparental mapping populations; GW = for QTL revealed in a genome-wide association (GWAS) study; RIL = recombinant inbred line.

****R2 (%) single value indicates the average QTL R2 across environments. A two-values range reports the QTL R2 range across environments.

^¤^Stable QTL detected in more than one population.

A suitable population for carotenoid analysis are the recombinant inbred lines (RILs) in advanced selfing generations, doubled haploid (DH) populations, or populations derived from backcrosses (Elouafi et al., 2001; Pozniak et al., 2007; Singh et al., 2009; Tsilo et al., 2011; Colasuonno et al., 2014).

When wheat germplasm, including cultivars, advanced breeding lines, or germplasm collections, are considered, mapping methods of genome-wide association study (GWAS) have been applied to link some marker haplotypes with trait expression (Vargas et al., 2016; Colasuonno et al., 2017a; Fiedler et al., 2017). The principle behind the method is to estimate correlations among the genotypes and the phenotypes in panels of lines, based on the linkage disequilibrium between the allelic variants of molecular markers and causal genes (Gupta et al., 2005; Bush and Moore, 2012). This approach has been the official method for many years. In the last decade, it has been extensively used for different traits owing to the availability of high numbers of DNA-based markers uniformly distributed in the genome (such as the high-density maps obtained from single nucleotide polymorphisms, SNPs, Wang et al., 2014 and Maccaferri et al., 2014) and the improvement of statistical tools. These included improved mixed models that effectively take into account the interfering panel population structure effects (Breseghello and Sorrells, 2006; Maccaferri et al., 2006; Zhang et al., 2010; Maccaferri et al., 2011; Lipka et al., 2012). Although several reports of SNP markers, associated to QTL for carotenoid pigments, have been published to date (**Table 1**), they mostly relied on linkage mapping and biparental RIL populations. Only four studies were deeply focused on SSR and SNP-based association mapping in durum wheat (Reimer et al., 2008; Colasuonno et al., 2017a; N’Diaye et al., 2017; Rosello et al., 2018), and only one considered haplotypes instead of single bi-allelic markers (N’Diaye et al., 2018).

## Carotenoid QTL Regions

**Figure 2:**
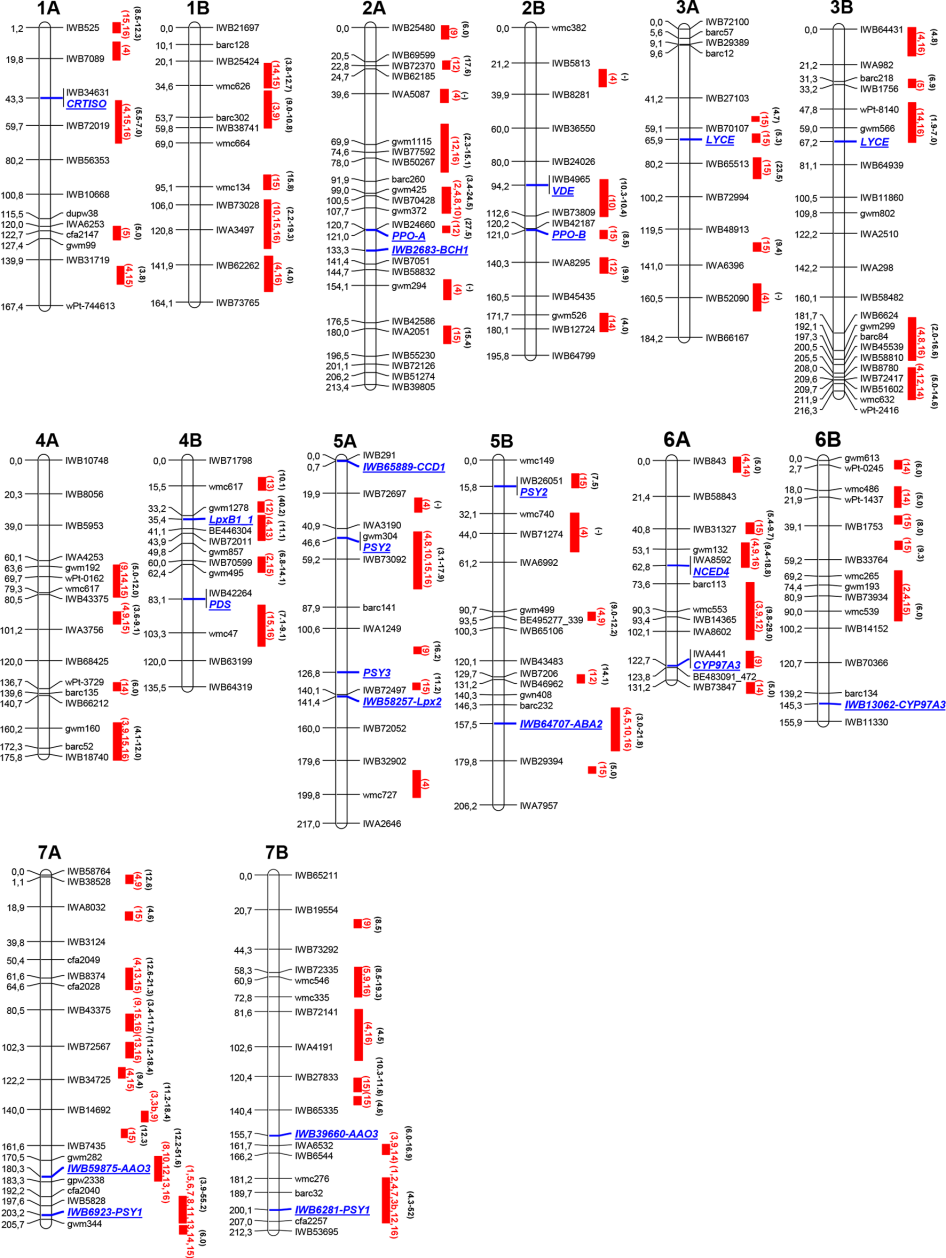
Schematic representation of durum wheat chromosomes [based on theMaccaferri et al. (2014) map] and quantitative trait loci (QTL) summary for yellow pigment content (YPC) or yellow index (YI) trait detected in references in **Table 1**. Markers, on the right chromosome side, are reported every 20 cM approximately. cM distances are indicated on the left side of the bar. Red solid bars indicate the QTL confidence interval regions. The red bars report the reference publication numbers in red (as in **Table 1** and **Table S1**) and the QTL R2 range value in black. 1, Elouafi et al. (2001); 2, Pozniak et al. (2007); 3, Zhang et al. (2008); 3b, Zhang and Dubcovsky (2008); 4, Reimer et al. (2008); 5, Patil et al. (2008); 6, Singh et al. (2009); 7, He et al. (2009); 8, Blanco et al. (2011); 9, Roncallo et al. (2012); 10, Colasuonno et al. (2014); 11, Campos et al. (2016); 12, N’Diaye et al. (2017); 13, Colasuonno et al. (2017a); 14, Rosello et al. (2018); 15, N’Diaye et al. (2018); 16, this study.

The results of recent peer-reviewed studies and additional studies provided by the authors reporting QTL for carotenoid pigments in durum wheat are compiled in **Table 1** (QTL clusters) and **Table S1** (all QTL), encompassing information on mapping population, examined phenotypic traits, and markers associated to carotenoid QTL. Map position of the major QTL clusters listed in **Tables 1** and **S1** are reported in **Figure 2**. The schematic representation is based upon the durum wheat consensus map published by Maccaferri et al. (2014), and revised for the carotenoid composition by Colasuonno et al. (2017a). Considering that each QTL/MTA identified by different studies was located in the same position of the durum wheat consensus map, we used the cM reported by Maccaferri et al. (2014) to identify the genome location.

Every chromosome is depicted with the first and the last SNP marker, and one marker every 20 cM. SSR markers have also been incorporated to anchor the actual consensus map with the previous ones. For most of the detected QTL, the connection between the different maps has been quite simple to be achieved.

The QTL mapping study highlights the differences in number and map position of the QTL identified in several analyses. This could be due to the specific plant material and/or the analytical and statistical procedures adopted in each study (e.g., linkage mapping vs. GWAS). In fact, the presence of numerous genes with an additive effect on the trait, the parental influence on the genotypes of the mapping populations, the genotype × environment interaction, and the number of markers used may affect the results. Moreover, a different carotenoid measurement approach and statistical procedures adopted might influence the reproducibility of each QTL analysis.

Mapping studies for YPC and YI identified 81 QTL (including singletons and QTL clusters) distributed on all wheat chromosomes (**Table S1**). Some of these QTL have been detected in more than one map/population, indicating the presence of stable alleles valuable for enhancing color and nutritional value of wheat grain (QTL clusters, listed in **Table 1**). Twenty stable QTL (highlighted in **Table 1**) were detected on chromosomes 1A (two), 1B (two), 2A, 2B, 3B, 4A (two), 4B, 5A, 5B (two), 6A, 6B, 7A (four), and 7B (two). Therefore, these QTL can be considered useful for MAS in breeding programs. Major, moderate, and minor QTL were defined basing on the percentage of the phenotypic variation (> 40%, 10–40%, and <10%, respectively) as reported in the different studies. The major QTL have been mapped on chromosomes 7AL (Elouafi et al., 2001; Patil et al., 2008; Zhang and Dubcovsky, 2008) and 7BL distal regions (Elouafi et al., 2001; Pozniak et al., 2007; Zhang and Dubcovsky, 2008). In particular, two different QTL have been identified on 7AL (Zhang and Dubcovsky, 2008; Blanco et al., 2011; Crawford and Francki, 2013): the first one (QTL-72) associated with allelic variations of the *AO* gene (Colasuonno et al., 2014; Colasuonno et al., 2017b) with a negative effect (R^2^ 22.1% for YPC and 18.4% for YI) on carotenoid content, and the second one (QTL-73) in the region of the *PSY1* gene (He et al., 2008; Zhang and Dubcovsky, 2008; He et al., 2009) with a positive and consistent effect up to 60% on YPC. The same QTL–gene associations are reported on chromosome 7BL with R^2^ up to 29.1% for the first QTL (QTL-80, at 180–189 cM) and 52% for the one at 181–211 cM (QTL-81). All the loci detected on chromosome group 7 resulted as negatively correlated with grain yield per spike (GYS) and thousand grain weights (TKWs) but positively correlated with protein content. The same association highlighted on chromosomes 7A and 7B between QTL and PSY2 gene has been reported on chromosome 5AS with moderate effect (R2 9.4–17.9% for YPC and 7.4–16.4 for YI) and negative correlation with GYS and TKW.

Various studies have confirmed the role of the violaxanthin de-epoxidase (VDE) on 2B (Tsilo et al., 2011; Roncallo et al., 2012) in YPC and YI content. The association between VDE and the QTL on 2BL at 94–113 cM has been validated by many results gained thanks to biparental mapping populations. Colasuonno et al. (2014) identified a QTL on 2BL at 94–113 cM with moderate and negative effect from the “Ciccio” alleles (16.4%).

Minor QTL have been detected on chromosomes 1A, 1B, 2A, 3A, 3B, 4A, 4B, 5A, 5B, and 6B (Pozniak et al., 2007; Zhang et al., 2008). Among them, 2A, 4B, and 6A showed variable effect with R^2^ of 3.5–15% for YPC and 12.3–21% for YI, 4–11.9% for YPC and 26.2 for YI, and 14–17% for YPC and 28.3% for YI, respectively (**Table 1** and **Table S1**). Lipoxygenase (*LpxB1.1*) gene is located on chromosome 4B, in the same site of the QTL described in literature by several authors (QTL-40; Carrera et al., 2007; Reimer et al., 2008; Verlotta et al., 2010; Colasuonno et al., 2017a).

QTL for individual carotenoid constituents in wheat were outlined by Howitt et al. (2009) and Blanco et al. (2011). Considering wheat rice synteny, two genes, phytoene synthase (*Psy-A1*) and ε-cyclase (*ε-LCY*), were identified by Howitt et al. (2009) as candidate genes for two of the QTL involved lutein content in wheat endosperm. A segregant population, achieved from crossing the durum wheat cultivars Primadur and Latino, was used by Blanco et al. (2011) to detect QTL for individual carotenoid compounds (lutein, zeaxanthin, β-cryptoxanthin, α-carotene, and β-carotene), YI, and yellow pigment concentration. Total carotenoid concentration accounted for 30–50% of the yellow pigment quantities in durum wheat (Hentschel et al., 2002), reflecting unknown color-producing substances in the durum extracts. Lutein was the most prominent carotenoid compound identified, followed by zeaxanthin, α-carotene, and β-carotene, whereas β-cryptoxanthin was a secondary component. QTL mapping identified clusters of QTL for total and/or one or more carotenoid compounds (α-carotene and β-carotene) in the same chromosomal zones (2A, 3B, 5A, and 7A) where QTL for yellow pigment concentration and YI were detected. The existence of molecular markers related to the main QTL previously indicated is a valuable tool for marker-assisted selection (MAS) programs to increase high carotenoid concentration and the nutritional value of wheat grains.

## Candidate Genes for Endosperm Yellowness

### Candidate Genes for Carotenoid Biosynthesis

The “candidate gene approach” has been used in QTL or association mapping to test SNPs within a candidate gene for a significant association with the yellow color character. Although most studies were focused in increasing the carotenoid content or altering the relative components through conventional breeding (Schulthess and Schwember, 2013), some carotenoid genes have been characterized and/or linked to QTL for carotenoids. In wheat, significant attention has been given to the carotenoid biosynthesis genes (*PSY* (Pozniak et al., 2007; He et al., 2008; Dibari et al., 2012; Campos et al., 2016; Vargas et al., 2016), lycopene ε-cyclase (*LCYE*) (Howitt et al., 2009; Crawford and Francki, 2013), lycopene β-cyclase (*LYCB*) (Zeng et al., 2015), carotenoid β-hydroxylase (*HYD*) (Qin et al., 2012), carotene desaturase (*PDS*), and *ZDS* (Cong et al., 2010), while the degradation of carotenoids has been studied by some catabolic genes, such as aldehyde oxidase (*AO*) (Colasuonno et al., 2017b), polyphenol oxidase (*PPO*) (Si et al., 2012), lipoxygenase (*LOX* or *LPX*) (Verlotta et al., 2010), and peroxidase (*PER*) (Asins and Perez de la Vega, 1985; Fraignier et al., 2000; Ficco et al., 2014).

As previously noted, the gene with major effect on YPC and YI traits is the *PSY*, which is essential for the carotenoid accumulation in the kernels (Gallagher et al., 2004). Phylogenetic approaches have identified three different *PSY* isoforms: *PSY1*, *PSY2*, and *PSY3* mapped on homoeologous chromosome groups 7, 5, and 3, respectively (Dibari et al., 2012). Atienza et al. (2007) was the first study to show that *PSY1* was located on chromosomes 7A and 7B of durum wheat. The gene coding for *PSY1* on chromosome 7B was found to be co-segregant with a carotenoid QTL, while the *PSY1* on chromosome 7A behaved as a co-dominant marker explaining 20–28% of the phenotypic variability (Pozniak et al., 2007). The effect of the *PSY1* loci on the endosperm yellowness of wheat is variable depending on the genetic background. Therefore, the percentage of explained variability of endosperm yellowness for *PSY1-A1* has been found ranging from medium (10–30%), to high (30–50%) and very high (> 50%) (Blanco et al., 2011; Colasuonno et al., 2014), while the reported values for *PSY1-B1* are from low (< 10%) to medium (10–30%) (Zhang and Dubcovsky, 2008; Roncallo et al., 2012) in durum wheat. Overall, the effect of alternative alleles of *PSY-A1* appears to be the most important in the variation of semolina yellowness (SY) using different durum wheat populations (Campos et al., 2016; Vargas et al., 2016). In these studies, *PSY1-Al* was associated to higher SY due to the allelic variant *l* of this gene. The use of molecular markers linked to *PSY1-A1* (i.e., Psy1-A1_STS and YP7A-2 studied jointly) in MAS was suitable to enhance grain yellow pigmentation (Campos et al., 2016). In addition, at 35 days after anthesis, *PSY1-A1* was 21-fold higher expressed in the high-yellowness compared to the low-yellowness genotypes evaluated, corroborating the major role of *PSY1-A1* in the genotypes associated to high SY (Vargas et al., 2016).

Additional evidence highlights how other genes are involved in the control of grain amber color. The full-length DNA sequence of a *ZDS* gene on wheat chromosome 2A, designated *Zds-A1*, was cloned, and a co-dominant functional marker, YP2A-1, was designed based on the polymorphisms of two alleles (Dong et al., 2012). The functional marker, explaining 11.3% of the phenotypic variance for YP content, was co-segregating with a QTL for YP content detected on chromosome 2A in a DH population.

The lycopene ε-cyclase gene (*LCYE*) associated to a QTL on chromosome 3A, playing an important role in the regulation of lutein content in wheat grain (Howitt et al., 2009). An SNP marker in *LCYE* was detected between two Australian wheat genotypes, and a highly significant (*P* < 0.01) association with a QTL on chromosome 3A in two mapping populations showed that *LCYE* is involved in color differences at a functional level (Crawford and Francki, 2013).

Zeng et al. (2015) were able to clone the lycopene β-cyclase gene (*LCYB*) and describe its function and connection with β-carotene biosynthesis in wheat grains. Their results suggest that *LCYB* has a key function in β-carotene biosynthesis in wheat and that *LCYB* may be useful to breed new wheat cultivars with high provitamin A content using RNA interference (RNAi) to hinder specific carotenogenic genes in the wheat endosperm.

Qin et al. (2012) characterized two genes, *HYD1* and *HYD2*, encoding β-hydroxylases in wheat. They observed different expression patterns for different *HYD* genes and homeologs in vegetative tissues and developing grains of tetraploid and hexaploid wheats, indicating their distinct regulatory control in tissue, grain development, and ploidy-specific fashions. The expression of *HYD*-*B1* achieved highest levels at the last stage of grain filling, showing how carotenoids were still synthesized in mature grains, raising the nutritional value of kernels.

In a recent investigation by Colasuonno et al. (2017), 24 candidate genes encompassed in the biosynthesis and catabolism of carotenoid compounds have been reported using wheat comparative genomics. SNPs identified in the coding sequences of 19 candidate genes enabled their chromosomal location and precise map positions on the two bread and durum reference consensus maps studied (Maccaferri et al., 2014; Wang et al., 2014). Six candidate genes (*PSY1, PSY2, CYP97A3, VDE, ABA2*, and *AAO3*) showing between one to five SNPs were significantly associated to YI by genome-wide association mapping in a collection of 233 accessions of tetraploid wheat, suggesting their involvement in the yellow pigment biosynthesis or catabolism. *PSY1, BCH1, CYP97A3, VDE*, and *ABA2* were also associated to YPC. The phenotypic variation (R^2^) explained by each of these markers ranged between 5.9% and 16.3% for YI and from 7.4% to 14.8% for YPC.

### Candidate Genes for Carotenoid Degradation

Lipoxygenase (*LPX*) genes, involved in the catabolic pathway, are the most characterized. LPX enzymes are non-heme iron-containing and dioxygenases are found in all kingdoms (Zhang et al., 2015), catalyzing the addition of oxygen in polyunsaturated fatty acids that possesses a cis, cis-1,4 pentadiene system (Garbus et al., 2009). In plants, lipoxygenases are found in leaves, seedlings, and seeds. LPX activity produces ROS that can induce oxidation and degradation of carotenoids (Borrelli et al., 2003). In durum wheat, there are different lipoxygenase genes and alleles contributing to the variation of pasta yellowness (Verlotta et al., 2010; Borrelli and Trono, 2016).

Holtman et al. (1996) reported that lipoxygenase-1 is responsible for LPX activity in barley seeds. Hessler et al. (2002) sequenced several wheat fragments, which were assigned to the *Lpx-1* locus based on their similarity to barley genes. De Simone et al. (2010) reported different levels of *Lpx-1* and *Lpx-3* transcripts at maturity between cultivars with contrasting LPX activities, whereas *Lpx-2* transcripts were absent at this stage. *Lpx-B1* locus was located on the short arm of chromosome 4B (Hessler et al., 2002; Garbus et al., 2009; Verlotta et al., 2010), and five related genes and allele sequences have been reported: *Lpx-B1.1a* (Genbank HM126466), *Lpx-B1.1b* (Genbank HM126468), and *Lpx-B1.1c* (Genbank HM126470) for the *Lpx-B1.1* locus, and *Lpx-B1.2* (Genbank HM126467) and *Lpx-B1.3* (Genbank HM126469) (Hessler et al., 2002; Carrera et al., 2007; Verlotta et al., 2010).

QTL analyses in durum wheat showed that 36–54% of the variation in LPX activity is explained by *Lpx-B1* (Carrera et al., 2007; Verlotta et al., 2010). Carrera et al. (2007) reported a deletion in the *Lpx-B1.1* gene, later named *Lpx-B1.1c* allele, which possesses a deletion covering from the second intron up to the last exon. This allele correlates with higher levels of pasta yellowness, due to the large deletion on its sequence, but it is not correlated with semolina or flour color, suggesting that the role of lipoxygenase on carotenoid degradation occurs in the pasta-making process rather than in the development of the grains (Carrera et al., 2007; Verlotta et al., 2010).

Verlotta et al. (2010) genotyped the presence of the *Lpx-B1.1* alleles in combination with either *Lpx-B1.2* or *Lpx-B1.3* in a diverse modern/old durum wheat population, and found three haplotypes: haplotype I (*Lpx-B1.3* and *Lpx-B1.1b*), haplotype II (*Lpx-B1.2* and *Lpx-B1.1a*), and haplotype III (*Lpx-B1.2* and *Lpx-B1.1c*), exhibiting high, intermediate, and low levels of functional *Lpx-B1* transcripts and enzymatic activity in mature grains, respectively, which is also correlated with β-carotene bleaching. Carrera et al. (2007) reported sequences corresponding to the *Lpx-2* and *Lpx-3* on chromosome group 5 and 4, respectively, and *Lpx-A3* showed significant effects on semolina color, but not on LPX mature grain activity, proving that the LPX activity given by Lpx-A3 acts earlier on grain development.

PPO (EC 1.14.18.1) catalyzes the oxidation of phenolic acids, producing short-chain polymers related to undesirable browning or darkening of pasta products, reducing its apparent quality (Watanabe et al., 2006). There are two non-linked genes controlling PPO activity on durum wheat that have been identified on the homoeologous chromosome 2A and 2B (Jimenez and Dubcovsky, 1999; Nair and Tomar, 2001; Simeone et al., 2002; Watanabe et al., 2004). Watanabe et al. (2006) reported that the locus on chromosome 2A was linked to PPO activity explaining 49.1% of the trait variation in an RIL population made from the crosses between Jennah Khetifa and Cham 1. Two *Ppo* paralogous families were mapped on the homoeologous group 2, named *Ppo-1* (*Ppo-A1* and *Ppo-B1*) and *Ppo-2* (*Ppo-A2* and *Ppo-B2*) (Beecher et al., 2012). *Ppo-A1* was found to have a major role in PPO activity (Simeone et al., 2002; Sun et al., 2005; Anderson et al., 2006; He et al., 2007). Using a marker developed for common wheat (PPO18) (Sun et al., 2005), four *Ppo-A1* alleles, named *Ppo-A1b*, *Ppo-A1f*, *Ppo-A1e*, and *Ppo-A1g*, were detected on durum wheat (He et al., 2009). Taranto et al. (2012) linked the different *Ppo-A1* alleles to distinct levels of PPO activity in 113 accessions of tetraploid wheat. *Ppo-A1f* was associated to high PPO activity, whereas *Ppo-A1b* and *Ppo-A1g* were related to low PPO activity, although they argued there was a consistent variability on the PPO activities associated with each allele. Using the reverse primer of marker PPO18 and a new forward primer specific for the *Ppo-A1* allele, Taranto et al. (2012) developed a new marker (MG18) able to detect the same alleles than PPO18 in a collection of 228 accessions of old, intermediate, and modern tetraploid wheats, but in a more efficient manner, reducing the variability on PPO activity of each allele-related group. Further decrease on PPO activity can be achieved by selecting also alleles for low PPO activity of the *Ppo-B1* and *Ppo-B2* paralogous genes, using markers MG08 and MG33, respectively, developed by Taranto et al. (2015). *Ppo-B1* and *Ppo-B2* were located 11 cM apart on chromosome arm 2BL. Marker MG08 identified four *Ppo-B1* alleles, related to high (*Ppo-B1c*) and low (*Ppo-B1a, Ppo-B1b*, and *Ppo-B1d*) PPO activity, whereas marker MG33 recognized two *Ppo-B2* alleles, associated to high (*Ppo-B2d*) and low (*Ppo-B2a*) PPO activity levels. The use of these markers in MAS breeding programs has the potential to further improve the color of pasta and durum wheat derived end products.

The aldehyde oxidase 3 (*AO-A3*) gene, located on chromosome 7AL, has been significantly associated to YI and to a QTL linked for YPC (Colasuonno et al., 2017b). Aldehyde oxidase enzymes (AO; EC 1.2.3.1) play roles in the final catalytic steps from carotenoids to ABA (Seo and Koshiba, 2002). qRT-PCR experiments revealed higher levels of *AO-A3* expression in the low YPC cultivar Ciccio in comparison to the high YPC cultivar Svevo. This gene also showed higher expression levels in the later stages of seed formation than *AO-A1* and *AO-A2*, suggesting a major role in the final stages of seed development (Colasuonno et al., 2017b). Colasuonno et al. (2017b) developed a marker for *AO-A3* for DHPLC, which could be useful for MAS programs.

Peroxidase (*PER*) genes have received less attention than the other carotenoid degradation genes on durum wheat, with most of the studies being conducted on common wheat, and no durum wheat specific markers for low peroxidase activity are available to date. Peroxidases (EC 1.11.1.7.) are oxidoreductases that oxidize a vast array of compounds present in plants, using hydrogen peroxide as substrate. They are related to pasta brownness due to the oxidation of phenolic substances. Studies showed that peroxidase genes are located on the homoeologous chromosome groups 1, 2, 3, 4, and 7 (Kobrehel and Feillet, 1975; Benito and de la Vega, 1979; Bosch et al., 1987; Liu et al., 1990; Wei et al., 2015). Up to 12 peroxidase isoforms are present in the durum grain, varying in quantity during kernel development, maturation, and germination, with some isoforms having specific locations between milling fractions (bran, semolina, and embryo), in which isoform P-5 is of importance because of its endosperm specific location, contributing to the darkening of pasta products (Feillet et al., 2000; Fraignier et al., 2000). Fortunately, PER do not show activity during pasta processing, likely due to the unavailability of hydrogen peroxide, its main substrate, whereas it is abundant in semolina (Feillet et al., 2000; Ficco et al., 2014).

## Novel Mutations in Carotenoid Genes

New advances in wheat genomics resources and in molecular technologies contributed to increase the knowledge of carotenoid genes. This included the screening of mutant resource containing chemically induced point mutation variation in candidate genes through TILLING strategy (Targeting Induced Local Lesions in Genomes) (Uauy et al., 2009; Colasuonno et al., 2016; Richaud et al., 2018).

Among all genes involved in the carotenoid pathway, LCYE and LCYB were the only genes extensively studied with this approach, highlighting the complexity of the trait and the difficulty of its modification.

Colasuonno et al. (2016) screened 1,140 mutant lines (0.70–0.85% ethyl methane sulfonate, EMS) focusing on these two target genes. The denaturing high-performance liquid chromatography (DHPLC) allowed to identify a total of 38 and 21 mutations for LCYE and LCYB genes, respectively, with a mutation density of 1/77 kb. Similarly, the analysis of 1,370 DNA individuals from the durum wheat Kronos TILLING mutant population (Uauy et al., 2009) allowed to identify 39 mutants for the LCYE homologues (Richaud et al., 2018) through CelI/agarose method.

Although in both studies, premature stop codons or deleterious missense mutations had been identified, no significant differences in the increment of β-carotene and total carotenoid content among the lines and the relative control were detected. This could be attributed to a high number of effective genes underlying this complex trait and their influence on the final phenotype.

Additional availability of an *in silico* TILLING population (Krasileva et al., 2017) allowed to identify knockout alleles in these target genes and give information about their mutation rate. For instance, of over 1,500 EMS mutagenized lines from the Kronos cultivar, 76 and 128 mutations had been detected in the LCYE and LCYB protein coding regions, respectively. Among all these mutations, only for LCYE gene 7 premature stop codons or deleterious missense mutations resulted to have significant effect on the change of protein composition. The low rate of deleterious SNPs for the target genes marked their main role into the biosynthesis and how some unknown mechanism prevented mutations in these key carotenoid enzymes.

Extensive studies specific for other carotenoid genes are needed to understand the available mutations and their potential effects if combined in double mutants on the final phenotype.

## Transfer of QTL or Genes Linked to High Ypc

Backcross breeding has been used to transfer gene(s) or QTL of interest from a certain genetic background into an elite cultivar lacking for carotenoids. Subsequently, MAS technology validated the additive effect of the locus/candidate gene and assessed its impact on the new genetic background (Hospital, 2005).

Even though there is a great number of works of QTL linked to high YPC identification (**Table 1**), direct validation on durum wheat and use through introgression is limited. Patil et al. (2018) developed a marker, PSY-1SSR, based on the microsatellite variations in the promoter region of Psy-1, allowing the identification of eight alleles of Psy-A1 and seven alleles of Psy-B1 simultaneously, linked to Qyp.macs-7A, a major QTL for YPC on the long arm of chromosome 7A identified in a PDW 233/Bhalegaon 4 RIL population (Patil et al., 2018). They used this marker to improve YPC through MAS of two different low YPC Indian cultivars, MACS 3125 and HI 8498, and they were able to follow the introgression of the allele Psy-A1SSRe (linked to high yellowness) from PDW 23, using backcross breeding. MACS 3125 backcrossed lines showed a significant increase in YPC (6.16–7.7 ppm) over the recurrent parent MACS 3125 (3.57 ppm). HI 8498 introgressed lines also showed a significant YPC increase (5.0–7.46 ppm) in comparison to their recurrent parent (3.26 ppm).

MAS is currently being used by CIMMYT and by the Canadian durum wheat breeding programs (Randhawa et al., 2013; N’Diaye et al., 2018) by selecting materials with low LOX activity, with the implementation of the LOXA marker (Carrera et al., 2007), targeting the Lpx-B1.1c allele (Verlotta et al., 2010) for the generation of breeding lines (Randhawa et al., 2013; Dreisigacker et al., 2016).

Even though there is a great number of works of QTL linked to high YPC identification (**Table 1**), direct validation on durum wheat and use through introgression is limited. Patil et al. (2018) developed a marker, PSY-1SSR, based on the microsatellite variations in the promoter region of *Psy-1*, allowing the identification of eight alleles of *Psy-A1* and seven alleles of *Psy-B1* simultaneously, linked to *Qyp.macs-7A*, a major QTL for YPC on the long arm of chromosome 7A identified in a PDW 233/Bhalegaon 4 RIL population (Patil et al., 2018). They used this marker to improve YPC through MAS of two different low YPC Indian cultivars, MACS 3125 and HI 8498, and they were able to follow the introgression of the allele *Psy-A1SSRe* (linked to high yellowness) from PDW 23, using backcross breeding. MACS 3125 backcrossed lines showed a significant increase in YPC (6.16–7.7 ppm) over the recurrent parent MACS 3125 (3.57 ppm). HI 8498 introgressed lines also showed a significant YPC increase (5.0–7.46 ppm) in comparison to their recurrent parent (3.26 ppm).

MAS is currently being used by CIMMYT and by the Canadian durum wheat breeding programs (Randhawa et al., 2013; N’Diaye et al., 2018) by selecting materials with low LOX activity, with the implementation of the LOXA marker (Carrera et al., 2007), targeting the *Lpx-B1.1c* allele (Verlotta et al., 2010) for the generation of breeding lines (Randhawa et al., 2013; Dreisigacker et al., 2016).

## Future Perspectives and Conclusions

Understanding the biosynthetic pathway for carotenoid pigment accumulation requires many efforts due to the durum wheat polyploidy and its quantitative nature. The information examined in this article explains the significant goals that have been reached in the last two decades in understanding the genetic and the molecular mechanisms underlying the metabolism and regulation of wheat carotenoids. Furthermore, the characterization of specific plant materials and the release of the durum wheat genome sequences, together with the development of more accurate classes of DNA-based markers and consensus maps, have allowed the identification of important genes involved in the control of carotenoid biosynthesis and catabolism.

Clearly, the most studied and repeatable QTL are those located on chromosomes 3AS (linked to the *LCYE* gene), 7AL, and 7BL (both tightly linked to the *PSY1* genes). Diagnostic markers are available in all these regions for MAS application. Hopefully, other carotenoid QTL regions will likely be further characterized in the future, taking advantage of the recent results and tools for identifying the candidate genes involved in the accumulation/degradation of the carotenoid compounds. This will certainly increase the speed of the genetic gains of carotenoid accumulation, which will benefit the breeding programs and the pasta industry. According to these new resources, we can anticipate an implementation in genotypic selection flanking the traditional phenotypic selection in the durum wheat breeding programs. At the same time, the additive effects of the genes involved in yellowness will generate improved plants through several breeding cycles able to incorporate the beneficial alleles introgressed. Future developments on MAS breeding will focus on selecting many genes alleles at once in order to reach such purpose. Despite all the research in this subject, efforts should be taken on the transfer of knowledge between the bench and the field, because of the current use of the markers described in this review that could potentially benefit the durum wheat breeding programs globally.

Finally, further emphasis of future activities will encompass the analysis of the genetic variability present in the durum wheat germplasm collections (i.e., pre-breeding work), and the TILLING populations, to better understand the functions of the genes involved in the structural and the regulatory system responsible for the YPC trait. Advanced techniques (i.e., CRISPR-Cas9–based genome editing method), will be useful if combined and used to understand the homoeologous silenced gene acting additively and imposing effects on both the total gene expression and the resulting phenotype. Taking these strategies together, the characterization of each gene could provide opportunities for diversifying the genetic architecture of carotenoid pigments and expand the existing allelic variation available for wheat improvement.

## Author Contributions

PC, AG, RP, and AS designed the review. MM, GC, RT, AB, IM, JA-F, and AC prepared the manuscript. All authors read and approved the final manuscript.

## Conflict of Interest

The authors declare that the research was conducted in the absence of any commercial or financial relationships that could be construed as a potential conflict of interest.
